# Measurement Properties of the Patient-Specific Functional Scale in Rehabilitation for Patients With Stroke: A Prospective Observational Study

**DOI:** 10.1093/ptj/pzad014

**Published:** 2023-02-13

**Authors:** Janne Evensen, Helene Lundgaard Soberg, Unni Sveen, Knut A Hestad, Jennifer L Moore, Berit Arnesveen Bronken

**Affiliations:** Department of Physical Medicine and Rehabilitation, Innlandet Hospital Trust, Gjøvik, Norway; Faculty of Health Sciences, Oslo Metropolitan University, Oslo, Norway; Department of Physical Medicine and Rehabilitation, Oslo University Hospital, Oslo, Norway; Faculty of Health Sciences, Oslo Metropolitan University, Oslo, Norway; Department of Physical Medicine and Rehabilitation, Oslo University Hospital, Oslo, Norway; Department of Mental Health and Rehabilitation, Faculty of Health- and Social Sciences, The Inland Norway University of Applied Sciences, Elverum, Norway; Department of Research, Innlandet Hospital Trust, Brumunddal, Norway; Regional Center of Knowledge Translation in Rehabilitation, Sunnaas Rehabilitation Hospital, Oslo/Nesodden, Norway; Department of Mental Health and Rehabilitation, Faculty of Health- and Social Sciences, The Inland Norway University of Applied Sciences, Elverum, Norway

**Keywords:** Goals, Patient-Reported Outcome Measure, Patient-Specific Functional Scale, Rehabilitation, Shared Decision Making, Stroke

## Abstract

**Objective:**

This study investigated the validity, reliability, responsiveness, and interpretability of the Patient-Specific Functional Scale (PSFS) in subacute stroke rehabilitation to determine its suitability to measure patient-identified rehabilitation goals.

**Methods:**

A prospective observational study was designed according to the checklist from Consensus-Based Standards for Selecting Health Measurement Instruments. Seventy-one patients diagnosed with stroke were recruited in the subacute phase from a rehabilitation unit in Norway. The International Classification of Functioning, Disability and Health was used to assess the content validity. Assessment of construct validity was based on hypotheses for correlation of the PSFS and comparator measurements. We assessed reliability by calculating the Intraclass Correlation Coefficient (ICC) (3.1) and the standard error of measurement. The assessment of responsiveness was based on hypotheses for the correlation of change scores between the PSFS and the comparator measurements. A receiver operating characteristic analysis was conducted to assess responsiveness. The smallest detectable change and minimal important change were calculated.

**Results:**

Eighty percent of the PSFS items were classified as activities and participation in the International Classification of Functioning, Disability and Health, indicating satisfactory content validity. The reliability was satisfactory with an ICC of 0.81 (95% CI = 0.69–0.89). The standard error of measurement was 0.70 point, and the smallest detectable change was 1.94 points. Five of 7 hypotheses were confirmed for construct validity, and 5 of 6 were confirmed for responsiveness, indicating moderate construct validity and high responsiveness. Assessing responsiveness with a criterion approach resulted in an area under the curve of 0.74. A ceiling effect was identified for 25% of the participants 3 months after discharge. The minimal important change was estimated to be 1.58 points.

**Conclusion:**

This study demonstrates satisfactory measurement properties for the PSFS in individuals undergoing inpatient stroke rehabilitation.

**Impact:**

This study supports the use of the PSFS to document and monitor patient-identified rehabilitation goals in patients receiving subacute stroke rehabilitation when applied using a shared decision approach.

## Introduction

Stroke remains a leading cause of adult disability.^1^^,^^2^ More than 50% of patients with a stroke have limitations in activities of daily living, and up to 40% use a manual wheelchair at rehabilitation discharge.^3^ Other challenges after a stroke include visual field loss (15%–52%) and dysphagia (42%–67%).^3^ More than one-half of individuals with stroke experience reduced cognitive function such as problems with memory, orientation, and attention.^4^ Further, 30% experience aphasia^5^ and 50% experience fatigue.^6^ However, evidence-based stroke rehabilitation can maximize recovery and improve quality of life.^3^ Rehabilitation facilitates the achievement of a person’s functional potential in their work and living environments. Health professionals should tailor rehabilitation interventions in collaboration with the patients to achieve each patient’s goals.^7^ Shared decision-making during the goal-setting process may increase motivation, confidence, and the sense of ownership of rehabilitation.^8^^,^^9^ Guidelines recommend that health professionals use standardized measurements to detect functional changes and evaluate the rehabilitation benefits for individuals with stroke.^10^

Patient-reported outcome measures capture patients’ self-reported functioning.^10^ These measurements are often predefined with standardized questions and replies, limiting a questionnaire’s relevance. Patient-specific measurements are a subcategory of patient-reported outcome measures that do not contain standardized questions. Instead, these measurements enable patients to identify their problems and current level of functioning using a rating scale.^11^ Hence, patient-specific measurements may be more specific to each patient’s functional problems than other standardized measurements.^12^^,^^13^ Further, these measurements require the active involvement of patients to identify and rate their problems.^11^ Moore et al developed a clinical practice guideline containing a core set of outcome measurements for adults with neurologic conditions (ie, an injury or disease to the central or peripheral nervous system) undergoing rehabilitation.^10^ The authors emphasized that health professionals should document patient-identified goals and monitor changes using a relevant outcome measurement.

The Patient-Specific Functional Scale (PSFS) is 1 of 11 patient-specific measurements used during goal setting^11^ and to document a patient’s problems during functional activities.^14^ First, the patients identify 1 to 5 activities (ie, PSFS items) in which they are experiencing difficulties because of injury or illness. Then, patients rate their current level of functioning associated with each activity on a numeric rating scale from 0 to 10, where 0 is “unable to perform the activity” and 10 is “performs the activity without difficulties or at the same level as before the injury/ illness.”^14^ The PSFS can be used across ages and levels of disability and by various professions across different levels of health services.^15^ It requires few resources for training and minimal equipment,^16^ is easy to administer, and is easily understood by patients.^15^ A Norwegian version of the PSFS was validated for patients with musculoskeletal disorders in primary care.^17^^,^^18^

Pathak et al^19^ used Consensus-Based Standards for Selecting Health Measurement Instruments (COSMIN) guidelines to assess measurement properties of the PSFS in a systematic review and found sufficient measurement properties in musculoskeletal disorders and some nonmusculoskeletal disorders. However, the authors emphasized the PSFS needs further evaluation before clinical use in health conditions such as stroke. Moore et al^10^ found that no patient-specific measurement had sufficient evidence across neurologic conditions to support a clinical practice guideline recommendation. The authors stated that the PSFS might be appropriate to administer but concluded that there were gaps in the literature regarding its measurement properties for these populations. Evensen et al^20^ investigated the applicability of the PSFS in patients with acquired brain injury (92% with stroke) and concluded that 92% (n = 54) could complete the PSFS. The patients who could not complete the PSFS (n = 5) demonstrated severe cognitive or language impairment.

A barrier to PSFS administration in patients with neurologic conditions is the potential impact of cognition, self-awareness, and language impairments.^10^^,^^21^^,^^22^ Heldmann et al^23^ reported that the PSFS has satisfactory measurement properties for assessing patient-specific functional limitations and changes in older patients with and without cognitive impairment hospitalized for acute stroke. The authors found good to excellent relative reliability, and construct validity was supported in both groups. Research on a similar patient-specific measurement, the Canadian Occupational Performance Measure,^24^ demonstrated excellent test–retest reliability and confirmed discriminant validity in patients with stroke.^22^ Although this demonstrates the potential to use patient-specific outcome measurements in stroke rehabilitation, the measurement properties of the PSFS have not been established in this diagnostic group.^10^^,^^19^

The literature demonstrates the need to identify a measurement that can document and monitor goals for patients undergoing stroke rehabilitation.^10^ Hence, this study investigated the validity, reliability, responsiveness, and interpretability of the PSFS in subacute stroke rehabilitation to determine its suitability to measure patient-identified rehabilitation goals.

## Methods

### Study Design, Participants, and Setting

This was a prospective observational study and was designed and reported according to the COSMIN checklist.^25–27^ Patients with stroke admitted to a specialized rehabilitation unit in a Norwegian regional hospital for ≥10 days were invited to participate. Inclusion criteria were <6 months after stroke, ability to communicate in Norwegian, and ability to provide informed consent. In addition, exclusion criteria were inability to perform the PSFS and the presence of progressive cancer or a progressive neurological disorder.

The patients were admitted to the rehabilitation unit within 6 months after stroke onset, and the mean length of stay in 2019 for patients with stroke was 13 days (range = 1–66). The PSFS was administered within 2 days of admission and discharge. In addition, a 3-month follow-up was completed after discharge (Tab. 1). The care team at the rehabilitation unit applied an interdisciplinary rehabilitation model, and a coordinator (ie, a nurse, occupational therapist, or physical therapist) organized the rehabilitation goal setting and activities during the stay. In collaboration with the patient, each coordinator applied the PSFS using a shared decision approach (example in Suppl. Appendix 1). Goal setting was guided by an evidence-based Norwegian guideline instructing how long-term goals can be transformed into specific and short-term goals and a PSFS activity.^28^ The patients received a written brochure about goal setting in a rehabilitation process. The coordinators attended training in utilizing the PSFS that included role-play exercises, observing a collaborative goal-setting process that involved PSFS completion, and supervision.

**Table 1:** Measurement Description, Scoring, and Assessment Timing

**Construct**	**Measure**	**Description and Scoring**	**Used For:**	**Assessment Time Point(s)** ^***a***^
Problems during functional activities	PSFS^14^	Patient-specific outcome measure scored on a numeric rating scale (0–10)	Measurement to be assessed	T1, T2, T3, T4
Stroke severity	National Institutes of Health Stroke Scale^36^^,^^62^	4 items on ocular movement, vision, speech, and language, scored on a 3- or 4-point ordinal scale	Medical characteristics	T1
Cognitive function	Montreal Cognitive Assessment^35^	11 items scored on a 1- to 5-point ordinal scale (0–30, worst to best); <26 identify impairments in cognition^63^	Medical characteristics	T1
Functional independence	Modified Rankin Scale^64^	Single-item questionnaire with a 7-point ordinal scale (0–6, best to worst)	Construct validity and responsiveness with construct approach	T1, T3
Independence in mobility and daily activities	Barthel Activities of Daily Living Index^31^	10 items containing activities of daily living, bowel and bladder control, transfer, and ambulation scored on a 3- or 4-point ordinal scale (0–100, worst to best)	Construct validity and responsiveness with construct approach	T1, T3
Ambulation and assistance required to walk	Functional Ambulation Categories^32^	Single-item questionnaire with a 6-point ordinal scale (0–5, worst to best)	Construct validity and responsiveness with construct approach	T1, T3
Comfortable gait speed	4-M Walk Test^33^^,^^34^^,^^37^	Speed to walk 4 m without acceleration or deceleration, measured in m/s	Construct validity and responsiveness with construct approach	T1, T3
Perceived change in function	Global Rating Scale^26^^,^^65^	Single-item questionnaire (scored 1–7) with response option of “much improved,” “improved,” “slightly improved,” “no change,” “slightly worsened,” “worsened,” or “much worsened”	Responsiveness with construct approach, subgroup analyses of responsiveness, and calculation of minimal important change	T2, T3, T4

^a^ PSFS = Patient-Specific Functional Scale; T1 = within 2 days of admission; T2 = 48 hours after T1; T3 = within 2 days of discharge; T4 = 3 months after discharge.

### Measurements and Data Collection

This study included the assessment of content validity, construct validity, reliability, and responsiveness of the PSFS. The comparator measurements modified Rankin Scale (mRS), Barthel Index for Activities of Daily Living (BI), Functional Ambulation Categories (FAC), and 4-M Walk Test (4MWT) measure construct areas that are commonly affected by stroke and targeted by rehabilitation interventions.^16^^,^^29^^,^^30^ The comparator measurements Montreal Cognitive Assessment (MoCA), National Institutes of Health Stroke Scale, and Global Rating Scale (GRS) and assessment time points are presented in detail in Table 1. All of the measurements have satisfactory measurement properties for patients with stroke.^30–37^

Health professionals from the interdisciplinary care team and the first author (J.E.) collected the data from January 2020 to December 2021. Because of the COVID-19 pandemic, we could not collect data from March to September 2020. Occupational therapists and the first author administered the MoCA and completed the measure’s official training.^38^ The physical therapists applying the 4MWT were trained in administrating the test. Medical and sociodemographic information were extracted from the medical record. Three months after discharge, the PSFS, mRS, FAC, and BI were administered in an in-person interview or by telephone by the first author (J.E.) because of the COVID-19 pandemic.

### Assessment of Measurement Properties According to COSMIN

#### Content Validity

We used The International Classification of Functioning, Disability and Health (ICF)^39^ to assess the correspondence between the rehabilitation goals represented by the PSFS items for each patient and the construct measured by the PSFS.^26^ The authors J.E. and B.A.B. independently linked goals into either body functions or activities and participation components.^40^ A third author (H.L.S.) was consulted to resolve disagreement. We linked the goals based on the meaningful units in the text and selected multiple codes when a goal contained more components.^40^^,^^41^ The content validity was considered satisfactory if 80% of the goals could be classified in the ICF activities and participation component. Examples of goals classified into the ICF main components “body functions” and “activities and participation” are presented in Supplementary Appendix 2.

#### Construct Validity

We established construct validity using hypotheses for correlation between the PSFS and comparators mRS, BI, FAC, and 4MWT. The hypotheses were based on previous studies^22^^,^^23^^,^^42–44^ and consensus among the investigators. Table 2 describes the hypotheses.

**Table 2:** Hypotheses, Justifications, and Results for Assessing Construct Validity and Responsiveness With a Construct Approach*^a^*

**Parameter Tested**	**Aspect of PSFS**	**Comparator**	**Hypotheses and Justifications**	**Result**	**Confirmed Hypotheses**
Construct validity	Total score (n = 71)	BI	Fair (*r* = 0.26–0.49) based on previous studies; PSFS and BI assess related constructs^23^^,^^42^	*r* = 0.27*^b^*	Yes
	Items similar to BI items (n = 58; 121 items)	BI	Fair but higher than with PSFS total score, because ADL items in PSFS and BI assess the same construct	*r* = 0.48*^b^*	Yes
	Total score (n = 57)	4MWT, gait speed	Fair (*r* = 0.26–0.49) based on previous studies; PSFS and 4MWT assess related constructs^23^^,^^42^	*r* = 0.16	No
	Items similar to gait function (n = 52; 62 items)	4MWT, gait speed	Fair but higher than with PSFS total score; gait function items in PSFS and 4MWT assess the same construct	*r* = 0.41*^b^*	Yes
	Total score (n = 71)	FAC	Fair (*r* = 0.26–0.49) based on previous studies; PSFS and FAC assess related constructs	*r* = 0.32*^b^*	Yes
	Items describing gait function (n = 52; 62 items)	FAC	Fair but higher than with PSFS total score; gait function items in PSFS and FAC assess the same construct	*r* = 0.32*^b^*	No
	Total score (n = 71)	mRS	Low (*r* < 0.25) because mRS assesses a related but different construct	*r* = −0.23*^b^*	Yes
Responsiveness	Change score	GRS	Fair correlation (*r* = 0.26–0.49) because GRS and PSFS assess the same construct^46^^,^^66^	*r* = 0.46*^b^*	Yes
	Change score	BI	Low correlation (*r* < 0.25) because BI assesses a dimension of functional problems that may not be relevant to all patients with stroke	*r* = 0.25*^b^*	No
	Change score	4MWT, gait speed	Low correlation (*r* < 0.25) because 4MWT assesses a dimension of functional problems that may not be relevant to all patients with stroke^42^	*r* = 0.12	Yes
	Change score	FAC	Low correlation (*r* < 0.25) because FAC assesses dimensions of functional problems that may not be relevant to all patients with stroke^42^	*r* = 0.12	Yes
	Change score	mRS	Fair correlation (*r* = 0.26–0.49) because mRS assesses a construct related to but differs from the construct that PSFS assesses^42^	*r* = 0.27*^b^*	Yes
	Change score	GRS, stable and improved subgroups	Expectation of significant difference in PSFS change score for the 2 subgroups (improved and stable) because GRS and PSFS assess the same construct	*p* = .003	Yes

^a^ ADL = activities of daily living; BI = Barthel ADL Index; FAC = Functional Ambulation Categories; GRS = Global Rating Scale; mRS = modified Rankin Scale; 4MWT = 4-M Walk Test; PSFS = Patient-Specific Functional Scale.

^b^ *p* < .05.

#### Reliability

The health professionals completed the test–retest reliability scoring on the PSFS 48 hours apart. To determine the stability of each patient’s symptoms at the retest, we used the GRS, asking each patient, “With respect to your stroke, how do you perceive your difficulties now compared with 48 hours ago?” Those who reported “slightly improved,” “no change,” or “slightly worsened” were categorized in the stable subgroup and were included in the test–retest analysis.

#### Responsiveness

We evaluated responsiveness using a criterion approach. First, categorizing the GRS scores at discharge and 3 months after discharge into either the improved or the stable subgroup. The improved subgroup consisted of participants who reported “improved” and “much improved.” The stable subgroup consisted of participants who reported “slightly improved” and “no change.”

A second method to evaluate responsiveness was based on hypotheses for correlations between changes in PSFS scores and comparator changes (ie, GRS, mRS, BI, 4MWT, FAC) and hypotheses concerning expected mean differences between changes in PSFS scores in the improved and stable subgroups.^45^ We formulated the hypotheses based on previous studies^42^^,^^46^ and consensus among the authors. Because the comparator measurements included items that may be irrelevant to some patients, we did not expect them to capture the same amount of change as the PSFS. Further, we did not expect the single-item instruments (ie, mRS, 4MWT, and FAC) to capture change to the same extent as the PSFS.^44^  Table 2 describes the hypotheses.

### Data Analysis

We tested normality with Q-Q plots, Shapiro–Wilk tests, and visual inspection for all groups and subgroups and analyzed the data using parametric and nonparametric approaches as indicated by the test results. Continuous data are presented using means and SDs, range, and median and interquartile range. The categorical variables are presented as frequencies and percentages. We calculated a PSFS mean score by dividing the sum of the ratings by the number of identified activities. Further, we calculated a mean PSFS score for items describing gait function and another PSFS mean score for items similar to the BI (eg, ADL, transfer, and ambulation). The mean difference between groups was assessed with an independent-sample *t* test. In addition, we assessed the mean difference in repeated measures with the paired-sample *t* test or Wilcoxon signed rank test. *P* ˂.05 was considered statistically significant.

Construct validity was assessed using the Spearman correlation coefficient: low (*r* < 0.25), fair (*r* = 0.26–0.49), moderate to high (*r* = 0.50–0.74), and high to excellent (*r* ≥ 0.75).^47^ The construct validity was considered to be high if <25% of the hypotheses were rejected, moderate if 25% to 50% were rejected, and poor if >50% were rejected.^26^

Fifty participants are recommended for the test–retest analysis.^26^ Assuming that 70% of the participants were stable, 70 participants were required to obtain 50 participants in the stable subgroup. Reliability was assessed using the ICC(3.1) (2-way mixed-model single measure) with 95% CIs.^26^^,^^48^ An ICC ≥0.70 was considered satisfactory.^27^^,^^49^ A Bland–Altman plot was used to examine the mean difference between test and retest scores and estimated an agreement interval, within which 95% of the differences fell.^50^ The standard error of measurement (SEM) was derived from the ICC and was calculated as follows: SEM = SD × √1 − *r*.^26^

Responsiveness based on hypotheses was quantified by the Spearman correlation coefficient: low (*r* ≤ 0.25), fair (*r* = 0.26–0.49), moderate to high (*r* = 0.50–0.74), and high to excellent (*r* ≥ 0.75).^47^ The responsiveness was considered high if <25% of the hypotheses were rejected, moderate if 25% to 50% were rejected, and poor if >50% were rejected.^26^ We assessed perceived recovery with the GRS as an anchor, dichotomized as improved and stable. The GRS anchor was acceptable if the results identified a minimum correlation of 0.30 between the PSFS change scores and the anchor.^51^ Receiver operating characteristic (ROC) analysis investigated the extent to which the PSFS discriminates between the 2 subgroups (stable and improved).^49^ We considered that an area under the curve (AUC) ≥0.70 would be satisfactory.^49^

We estimated the smallest detectable change at the 95% confidence level (SDC_95_) as follows: SDC_95_ = 1.96 × √2 ×  SEM.^49^ The minimal important change was estimated using an anchor-based approach and an ROC. The optimal ROC cutoff point identified the minimal important change value.^52^ PSFS ceiling and floor effects were considering present if >15% of the participants achieved the minimum or maximum score.

The missing data are reported in the Results section. We used IBM SPSS version 28 (IBM SPSS, Armonk, NY, USA) for the statistical analysis.

### Role of the Funding Source

The funders had no role in the study’s design, conduct, or reporting.

## Results

### Participants

A total of 107 patients were assessed for inclusion. Twenty-one were excluded due to severe aphasia (n = 15), inability to speak Norwegian (n = 2), or inability to complete the PSFS for other reasons (n = 4). Six patients were excluded because their stroke onset occurred >180 days before admission. Nine declined to participate. Hence, the total sample was 71 patients. Seven participants were lost at the 3-month follow-up, and <5% of the data were missing because of practical data collection challenges for the health professionals. The missing data included a PSFS score at retest and 1 at 3 months after discharge. Two GRS scores were missing at retest, and 1 was missing at discharge. Table 3 presents participants’ characteristics at admission.

**Table 3:** Sociodemographic and Medical Characteristics of 71 Participants at Admission

Characteristic	Value*^a^*
Men/women	46 (65)/25 (35)
Age, mean (SD), y	71 (11)
Discharged to:	
Home	52 (73)
Nursing home	15 (21)
Other places	4 (6)
Education level, y	
≤12	53 (75)
≥13	18 (25)
Length of stay in rehabilitation unit, mean (SD), d	17 (5)
Days after stroke, mean (SD)	31 (39)
≤6	9 (13)
7–89	57 (80)
90–180	5 (7)
Stroke location or type	
Ischemic stroke, right side	22 (31)
Ischemic stroke, left side	23 (32)
Hemorrhagic stroke	9 (13)
Cerebellar stroke	2 (3)
Brain stem stroke	6 (8)
Unclassified	9 (13)
Aphasia	20 (28)

^a^ Data are reported as number (percentage) of participants unless otherwise indicated.

The PSFS mean scores were normally distributed, but the BI and MoCA scores were not. The PSFS mean scores were 3.8 (SD = 1.6) at admission, 6.6 (SD = 2.2) at discharge, and 7.9 (SD = 1.9) at the 3-month follow-up. The PSFS mean score improved by a mean of 2.6 (SD = 2.0) points from admission to discharge and by a mean of 1.2 (SD = 1.8) points from discharge to the 3-month follow-up. The median admission MoCA score was 20 (interquartile range = 17–25) points, and 84% of participants had cognitive impairments. Table 4 describes the test results.

**Table 4:** Results at Admission and Discharge and Change Scores from Admission to Discharge*^a^*

**Measurement**	**Value at:**		**Change Score**
	**Admission**	**Discharge**	
PSFS, mean (SD)	4.0 (1.8)	6.6 (2.2)	2.6 (2)*^b^*
mRS, median (IQR)	4 (3–4)	3 (2–4)	0 (−1 to 0)*^b^*
BI, median (IQR)	74 (60–95)	83 (70–100)	5 (0 to 15)*^b^*
FAC, median (IQR)	3 (3–4)	4 (3–5)	1 (0 to 1)*^b^*
Walk without assistance/alone, no. (%) of participants	29 (41)	50 (70)	
Walk with assistance, no. (%) of participants	42 (59)	21 (30)	
4MWT, mean (SD), m/s	0.79 (0.32)	0.90 (0.28)	0.11 (0.23)*^b^*
Unable to walk in 4MWT without physical assistance, no. (%) of participants	15 (21)	7 (10)	
MoCA total score, median (IQR)	20 (17–25)	*^d^*	
Cognitive impairments of <26 points on MoCA, no. (%) of participants	58 (84)*^c^*		

^a^ BI = Barthel Activities of Daily Living Index; FAC = Functional Ambulation Categories; MoCA = Montreal Cognitive Assessment; mRS = modified Rankin Scale; 4MWT = 4-M Walk Test; PSFS = Patient-Specific Functional Scale.

^b^ *P* < .05.

^c^ n = 69.

^d^ Not used at discharge (T3) because of a short time space between admission (T1) and discharge (T3) to avoid a learning effect.

### Content Validity

The participants (n = 71) identified 232 PSFS items with a median of 3 PSFS items per patient (range = 1–5). The content validity was satisfactory because 80% of the PSFS items were classified as activities and participation in the ICF.

### Construct Validity

The results demonstrated a fair to low correlation between the PSFS admission score and the comparator measurements. As shown in Table 2, we confirmed 5 of the 7 hypotheses (71%), indicating moderate construct validity.

### Reliability

**Figure 1:**
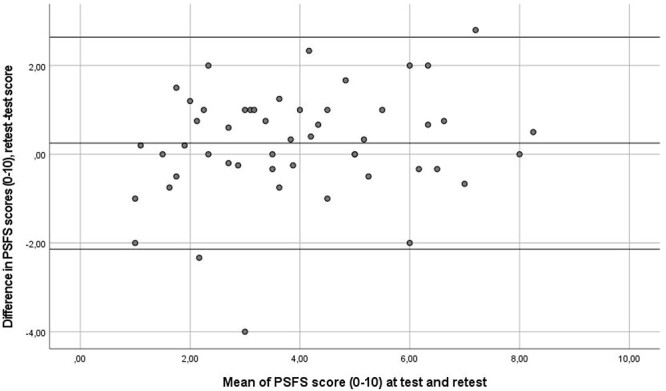
Bland–Altman plot of the Patient-Specific Functional Scale (PSFS) scores showing the mean difference between test and retest and constructing limits of agreement to estimate an agreement interval, within which 95% of the differences fall. The horizontal line in the middle represents the mean of the differences (d) between test and retest scores. The upper and lower lines indicate the 95% limits of agreement, obtained as d ± 1.96 SD of d.

The stable subgroup consisted of 51 participants, and 28 (55%) reported “slightly improved.” There was a significant change (*P* < .001) in PSFS mean score for the stable subgroup with a higher retest score of 0.25 (SD = 1.2) point. The ICC was 0.81 (95% CI = 0.69–0.89), and the SEM was 0.70 point. As shown in Figure 1, no systematic variability was demonstrated in a Bland–Altman plot, with 95% limits of agreement being −2.15 and 2.60 points.

### Responsiveness

The correlations between mean PSFS and comparator measurements’ change scores were low to fair. We identified statistically significant differences (*p* = .003) when comparing the mean PSFS score for the stable subgroup (1.49 points) with the improved subgroup (3.11 points). We rejected 1 of 6 hypotheses (17%), and the responsiveness was considered high (Tab. 2).

According to the GRS, 50 of the participants (71%) improved their scores, and 20 (29%) were stable from admission to discharge. The PSFS discriminated between participants in the stable subgroup and those in the improved subgroup with an AUC of 0.74 (95% CI = 0.61–0.87). Further, responsiveness testing from discharge to the 3-month follow-up identified 45 participants (70%) in the improved subgroup and 19 participants (30%) in the stable subgroup. The AUC was 0.30 (95% CI = 0.16–0.45), indicating low responsiveness for this period.

### Interpretability

**Figure 2:**
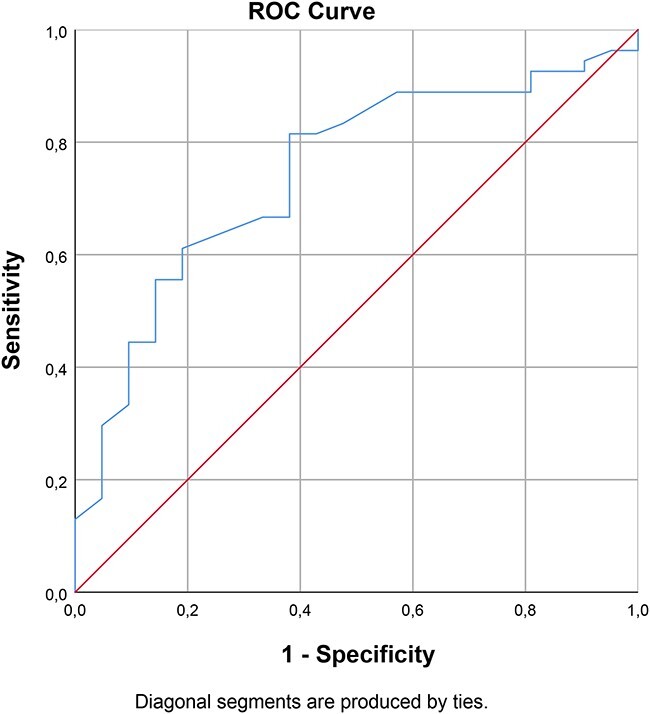
Receiver operating characteristic (ROC) was used to estimate the minimal important change by identifying the optimal cutoff point between the improved and stable subgroup.

In this study, no participants scored zero on the PSFS items at any time point or 10 at admission. Four participants scored 10 at discharge. At 3 months after discharge, 16 of 64 participants (25%) scored 10, indicating a ceiling effect at this time point. The SDC_95_ was 1.94 points. The correlation between the PSFS and the GRS change scores was 0.46 (*p*< .001); hence, the GRS was an acceptable minimal important change anchor. Figure 2 illustrates the optimal cutoff value of 1.58 in the ROC curve with a sensitivity of 0.82 and a specificity of 0.60. Hence, the minimal important change from admission to discharge was estimated to be 1.58 points.

## Discussion

This study supports the use of PSFS for goal setting in patients undergoing stroke rehabilitation. The results showed that the content validity, construct validity, and relative reliability (ICC = 0.81) were satisfactory. Further, the SEM and SDC_95_ were 0.70 and 1.94 points, respectively. The results indicated high responsiveness, and the PSFS discriminated between participants in the stable subgroup and those in the improved subgroup with an AUC of 0.74. The minimal important change was estimated to be 1.58 points.

### Content Validity

Eighty percent of the PSFS items were classified as activities and participation in the ICF, which is less than the number in this domain in a study on musculoskeletal disorders (median = 95%).^19^ Individuals with subacute stroke may be more concerned about their impairments and sudden loss of function than individuals with musculoskeletal disorders may be.^53^ In patients with traumatic brain injury and dizziness and balance problems, the PSFS items were classified as 65% activities and participation and 31% body functions.^54^ The amount of PSFS items classified as activities and participation is lower than in the present study (80%), which may reflect that dizziness and balance problems more often are classified as body function.

### Construct Validity

We confirmed 5 of 7 construct validity hypotheses. The correlation between gait speed assessed with 4MWT and the PSFS total score was lower than expected (*r* = 0.16). This conflicts with research identifying several associations between walking speed and functional tasks.^34^ Individuals with stroke often report challenges such as visual field loss, dysphagia, reduced cognitive function, and aphasia, which may influence the PSFS total score and reduce the correlation with gait speed. Fifty-nine percent of the participants in this study needed assistance for walking, and patient goals likely focused more on walking ability than walking speed.

The fair correlation between the PSFS and the comparators may indicate that the PSFS captures other aspects of functioning than the standardized measurements. The patient-centered nature of the PSFS that requires the exploration of individual needs may explain this finding. Thus, the PSFS may complement standardized measurements to provide a more comprehensive and patient-centered representation of functional problems for patients with stroke.

### Reliability

The relative reliability (ICC = 0.81) is consistent with PSFS studies in patients with musculoskeletal disorders (ICC = 0.55–0.98).^19^ Patients included in our study were admitted to rehabilitation ≤180 days after the stroke onset when poststroke recovery is rapid.^55^^,^^56^ In our research, the PSFS mean score was 0.25 point higher at retest than at test, which indicates an improvement for some of the participants over 48 hours. In addition, 55% of participants who were categorized as stable reported they improved slightly on the GRS at retest. An acute care study of older adults with and without cognitive impairments acknowledged a known rapid change in function during the first days.^23^ Therefore, the study investigators used a 24-hour period for assessing the test–retest reliability. Despite a shorter time from test to retest, the ICC was lower (0.76) than in the present study.^23^

Some authors recommend selecting measures with an ICC >0.90 when the measure guides decisions about an individual’s care, whereas an ICC >0.70 is acceptable when applied to group-level changes.^57^ For example, de Vet et al^26^ considered an ICC of 0.70 acceptable but values >0.80 or 0.90 to be more beneficial. Our study resulted in an ICC of 0.81 when trained health professionals administered the test. These data suggest that when applying the PSFS in clinical practice, training and minimizing sources for error will be critical to ensure reliable measurement.

In this study, the SEM accounts for the possibility that a change of 0.70 point may be due to random error. This finding is consistent with previous studies of patients with musculoskeletal disorders (SEM = 0.35–1.50)^19^ and older patients in an acute care setting with and without cognitive impairment (SEM = 0.78 and 0.83).^23^

### Responsiveness

We did not confirm the hypothesis regarding a low correlation between the PSFS and the BI change scores. Instead, the correlation was 0.25, which was just above the criterion. In this study, 58 of 71 participants identified PSFS items similar to items in the BI, indicating many participants have problems with activities of daily living and ambulation. Hence, we slightly underestimated this correlation in the a priori hypothesis.

These data indicate that the PSFS discriminates between participants in the stable subgroup and those in the improved subgroup with an AUC of 0.74. This finding aligns with the results of a systematic review that identified AUCs ranging from 0.61 to 0.99.^19^ In these studies, the follow-up period ranged from 6 days to 3 months, and the mean length of stay in the present study was 17 days. However, we identified low PSFS responsiveness between discharge and 3 months after discharge. The ceiling effect we identified 3 months after discharge may have hampered the capacity to capture improvement using the same PSFS items across admission, discharge, and follow-up.^26^ Hence, it may be optimal to consider new goals after discharge from inpatient rehabilitation.^58^^,^^59^

### Interpretability

The high scores in the PSFS at 3 months after discharge may indicate that the participants reached their preferred functional level. Therefore, the high scores might have been appropriate for the respective PSFS items.^26^ However, this might also reflect a need for reassessment and identifying new and more challenging goals throughout the rehabilitation process.

Based on these results, health professionals can consider a PSFS total score change of 2 points (SDC_95_) to reflect a change beyond measurement error for patients with subacute stroke. Similarly, the SDC_95_ values were 1.80 and 1.90 points for older patients with and without cognitive impairments and hospitalized for acute stroke, respectively, and 0.64 to 3.30 for individuals with musculoskeletal disorders.^23^ In the present study, the minimal important change was 1.58 points, which is similar to the minimal important change range of 0.80 to 2.90 points identified in other studies.^19^^,^^23^

### Implications for Practice

Disturbances of cognition, self-awareness, and language impairment in patients with stroke are considered a barrier to ascertaining the patients’ goals and ratings of the goals.^10^^,^^22^ In this study, 84% of the participants had cognitive impairments and 26% had aphasia. These results support the use of the PSFS in patients with stroke in a subacute stroke rehabilitation setting, even when language or other cognitive impairments are present. Another study involving patients with subacute stroke showed that 8% of the sample who were unable to complete the PSFS had severe cognitive or language impairment.^20^ Nevertheless, health professionals should exercise caution when using PSFS results to guide decision-making when patients have poor cognitive function or language difficulties.

In this study, the PSFS was administered within 2 days of admission and discharge, which allowed us to capture changes throughout the length of stay. As such, we recommend administering the PSFS at these time points to ensure all possible changes are captured during a patient’s stay. We also identified low responsiveness during the 3-month follow-up and recommend asking patients to identify new goals for periods after discharge from inpatient rehabilitation.

The SDC_95_ (1.94 points) in the present study was higher than the minimal important change (1.58 points). When applying the minimal important change to patients in practice, we recommend using a value ≥2.0 points to account for the SDC_95_. At a group level, achieving the SDC_95_ would imply that minimal important change was achieved. However, the threshold for the SDC_95_ should be exceeded when used with individuals or aggregated data. The PSFS was applied using a shared decision approach, and we recommend using similar methods when administering the measure in practice and research. These results may not be generalizable to other methods, such as asking patients to self-administer the PSFS.^26^

The scope of this study was to investigate the PSFS measurement properties when administered using a shared decision approach. Other clinical aspects that may be important for future research include the impact of the PSFS on the collaboration between the multidisciplinary team and patients’ families.

### Strengths and Limitations

Using COSMIN as a guide helped us design the study of the PSFS on the measurement properties. However, this also created a few challenges. Specifically, it was difficult to define a priori hypotheses including correlations for the construct validity analyses and responsiveness with a construct approach. Although COSMIN recommends these approaches, we found the literature that used these approaches to be scarce.

Because of the COVID-19 pandemic, we revised our initial data collection plan for the 3-month in-person follow-up. Regarding the PSFS, mRS, FAC, and BI, the first author (J.E.) collected the data in person or by telephone, which might have resulted in bias. However, in a meta-analysis, Rutherford et al^60^ reported that mixed modes of tests administration do not cause bias and may be a helpful strategy for reducing missing data.

Ideally, the percentage of participants in the improved or stable subgroup should be 50% to estimate minimal important change using an ROC analysis.^61^ Terluin et al^61^ found that minimal important change was estimated higher when the proportion improved was >50%. Further, de Vet et al reported that at least 35 participants are needed in each group to assess responsiveness with a construct approach.^26^ The stable and improved subgroups consisted of 20 and 50 in this study.^26^ Hence, the minimal important change value and responsiveness with a criterion approach should be interpreted with caution.^52^

This study demonstrates satisfactory measurement properties for the PSFS in individuals undergoing subacute stroke inpatient rehabilitation when applied using a shared decision approach. These data support using the PSFS to document and monitor patient-identified rehabilitation goals.

## Supplementary Material

Supplementary_Appendix_TSR_pzad014

## Data Availability

Because of the sensitive nature of the data collected for this study, as well as consent form restriction and health privacy laws, we are unable to provide public access to the data set.

## Presentation

This study was presented at the 2022 Rehabilitation Conference Helse Sør-Øst; September 14, 2022; Norway.

## References

[1] Stinear CM, Lang CE, Zeiler S, Byblow WD. Advances and challenges in stroke rehabilitation. Lancet Neurol. 2020;19:348–360. 10.1016/S1474-4422(19)30415-6. 32004440

[2] Global, regional, and national burden of stroke and its risk factors, 1990-2019: a systematic analysis for the global burden of disease study 2019. Lancet Neurol. 2021;20:795–820. 10.1016/S1474-4422(21)00252-0. 34487721 PMC8443449

[3] Winstein CJ, Stein J, Arena R et al. Guidelines for adult stroke rehabilitation and recovery: a guideline for healthcare professionals from the American Heart Association/American Stroke Association. Stroke. 2016;47:e98–e169. 10.1161/STR.0000000000000098. 27145936

[4] Barbay M, Diouf M, Roussel M, Godefroy O, GRECOGVASC Study Group. Systematic review and meta-analysis of prevalence in post-stroke neurocognitive disorders in hospital-based studies. Dement Geriatr Cogn Disord. 2018;46:322–334. 10.1159/000492920. 30504699

[5] Flowers HL, Skoretz SA, Silver FL et al. Poststroke aphasia frequency, recovery, and outcomes: a systematic review and meta-analysis. Arch Phys Med Rehabil. 2016;97:2188–2201.e8. 10.1016/j.apmr.2016.03.006. 27063364

[6] Cumming TB, Packer M, Kramer SF, English C. The prevalence of fatigue after stroke: a systematic review and meta-analysis. Inter J Stroke. 2016;11:968–977. 10.1177/1747493016669861. 27703065

[7] World Health Organization . Rehabilitation in health systems. Geneva: World Health Organization; 2017: Report No.: 978-92-4-154997-4, Contract No.: 18.12.18.

[8] Levack WM, Weatherall M, Hay-Smith EJ et al. Goal setting and strategies to enhance goal pursuit for adults with acquired disability participating in rehabilitation. The Cochrane Database Sys Rev. 2015;7:Cd009727. 10.1002/14651858.CD009727.pub2. PMC8941379 26189709

[9] Rose A, Rosewilliam S, Soundy A. Shared decision making within goal setting in rehabilitation settings: a systematic review. Patient Educ Couns. 2017;100:65–75. 10.1016/j.pec.2016.07.030. 27486052

[10] Moore JL, Potter K, Blankshain K, Kaplan SL, O'Dwyer LC, Sullivan JE. A core set of outcome measures for adults with neurologic conditions undergoing rehabilitation: a clinical practice guideline. J Neurol Phys Ther. 2018;42:174–220. 10.1097/NPT.0000000000000229. 29901487 PMC6023606

[11] Stevens A, Beurskens A, Koke A, van der Weijden T. The use of patient-specific measurement instruments in the process of goal-setting: a systematic review of available instruments and their feasibility. Clin Rehabil. 2013;27:1005–1019. 10.1177/0269215513490178. 23881336

[12] Hall AM, Maher CG, Latimer J, Ferreira ML, Costa LO. The patient-specific functional scale is more responsive than the Roland Morris disability questionnaire when activity limitation is low. Euro Spine J. 2011;20:79–86. 10.1007/s00586-010-1521-8. PMC3036014 20628767

[13] Pengel LH, Refshauge KM, Maher CG. Responsiveness of pain, disability, and physical impairment outcomes in patients with low back pain. Spine. 2004;29:879–883. 10.1097/00007632-200404150-00011. 15082988

[14] Stratford P, Gill C, Westaway M, Binkley J. Assessing disability and change on individual patients: a report of a patient specific measure. Physiotherapy Can. 1995;47:258–263.

[15] Barker RN, Sealey CJ, Polley ML, Mervin MC, Comans T. Impact of a person-centred community rehabilitation service on outcomes for individuals with a neurological condition. Disabil Rehabil. 2017;39:1136–1142. 10.1080/09638288.2016.1185803. 27281692

[16] Shirley Ryan Ability Lab . Rehabilitation measures database; 2013. Accessed March 1, 2022. https://www.sralab.org/rehabilitation-measures/database.

[17] Grotle M, Kjeken I. Patient-Specific Functional Scale, Norwegian translation. 1996. Unpublished.

[18] Moseng T, Tveter AT, Holm I, Dagfinnrud H. Pasient-Spesifikk Funksjons Skala—et nyttig verktøy for fysioterapeuter i primærhelsetjenesten. Norwegian J Physiother. 2013;2.

[19] Pathak A, Wilson R, Sharma S et al. Measurement properties of the patient-specific functional scale and its current uses: an updated systematic review of 57 studies using COSMIN guidelines. J Orthop Sports Phys Ther. 2022;52:262–275. 10.2519/jospt.2022.10727. 35128944

[20] Evensen J, Soberg HL, Sveen U, Hestad KA, Bronken BA. The applicability of the patient-specific functional scale (PSFS) in rehabilitation for patients with acquired brain injury (ABI)—a cohort study. J Multidiscip Healthc. 2020;13:1121–1132. 10.2147/JMDH.S259151. 33116558 PMC7553661

[21] Mathis RA, Taylor JD, Odom BH, Lairamore C. Reliability and validity of the patient-specific functional scale in community-dwelling older adults. J Geriatr Phys Ther. 2001 2019;42:e67–e72. 10.1519/JPT.0000000000000188. 29630006

[22] Yang SY, Lin CY, Lee YC, Chang JH. The Canadian Occupational Performance Measure for patients with stroke: a systematic review. J Phys Ther Sci. 2017;29:548–555. 10.1589/jpts.29.548. 28356652 PMC5361031

[23] Heldmann P, Hummel S, Bauknecht L, Bauer JM, Werner C. Construct validity, test-retest reliability, sensitivity to change, and feasibility of the patient-specific functional scale in acutely hospitalized older patients with and without cognitive impairment. J Geriatr Phys Ther. 2022;45:134–144. doi: 10.1519/JPT. 0000000000000303. 33734156 10.1519/JPT.0000000000000303

[24] Law M, Baptiste S, McColl M, Opzoomer A, Polatajko H, Pollock N. The Canadian Occupational Performance Measure: an outcome measure for occupational therapy. Canad J Occup Ther. 1990;57:82–87. 10.1177/000841749005700207. 10104738

[25] Mokkink LB, Terwee CB, Patrick DL et al. The COSMIN study reached international consensus on taxonomy, terminology, and definitions of measurement properties for health-related patient-reported outcomes. J Clin Epidemiol. 2010;63:737–745. 10.1016/j.jclinepi.2010.02.006. 20494804

[26] de Vet HC, Knol DL, Terwee CB, Mokkink LB. Measurement in Medicine: A Practical Guide. Cambridge: Cambridge University Press; 2010. 10.1017/CBO9780511996214.

[27] Mokkink LB, Prinsen CAC, Patrick DL et al. COSMIN Study Design Checklist for Patient-Reported Outcome Measurement Instruments. Department of Epidemiology and Biostatistics, Amsterdam Public Health Research Institute; 2019.

[28] Evensen J, Skøien R, Sareneva E et al. Rehabilitering. Samvalg i målavklaringsprosessen; Kunnskapsbasert fagprosedyre. Innlandet Hospital Trust, ed. Helsebiblioteket; 2015.

[29] Taylor-Rowan M, Wilson A, Dawson J, Quinn TJ. Functional assessment for acute stroke trials: properties, analysis, and application. Front Neurol. 2018;9:191. 10.3389/fneur.2018.00191. 29632511 PMC5879151

[30] Banks JL, Marotta CA. Outcomes validity and reliability of the modified Rankin scale: implications for stroke clinical trials: a literature review and synthesis. Stroke. 2007;38:1091–1096. 10.1161/01.STR.0000258355.23810.c6. 17272767

[31] Mahoney FI, Barthel DW. Functional evaluation: the Barthel index. Md State Med J. 1965;14:61–65. 14258950

[32] Mehrholz J, Wagner K, Rutte K, Meissner D, Pohl M. Predictive validity and responsiveness of the functional ambulation category in hemiparetic patients after stroke. Arch Phys Med Rehabil. 2007;88:1314–1319. 10.1016/j.apmr.2007.06.764. 17908575

[33] Guralnik JM, Ferrucci L, Pieper CF et al. Lower extremity function and subsequent disability: consistency across studies, predictive models, and value of gait speed alone compared with the short physical performance battery. J Gerontol A Biol Sci Med Sci. 2000;55:M221–M231. 10.1093/gerona/55.4.M221. 10811152 PMC12149745

[34] Middleton A, Fritz SL, Lusardi M. Walking speed: the functional vital sign. J Aging Phys Act. 2015;23:314–322. 10.1123/japa.2013-0236. 24812254 PMC4254896

[35] Toglia J, Fitzgerald KA, O'Dell MW, Mastrogiovanni AR, Lin CD. The Mini-Mental State Examination and Montreal Cognitive Assessment in persons with mild subacute stroke: relationship to functional outcome. Arch Phys Med Rehabil. 2011;92:792–798. 10.1016/j.apmr.2010.12.034. 21530727

[36] Lyden P, Lu M, Jackson C et al. Underlying structure of the National Institutes of Health stroke scale: results of a factor analysis. NINDS tPA Stroke Trial Investigators Stroke. 1999;30:2347–2354. 10.1161/01.STR.30.11.2347. 10548669

[37] Cheng DK, Dagenais M, Alsbury-Nealy K et al. Distance-limited walk tests post-stroke: a systematic review of measurement properties. NeuroRehabilitation. 2021;48:413–439. 10.3233/NRE-210026. 33967070 PMC8293643

[38] Nasreddine Z. MoCA Montreal Cognitive Assessment; 2021. Accessed March 1, 2022. https://www.mocatest.org/faq/

[39] World Health Organization . World Health Organization. International Classification of Function Disability and Health: ICF: World Health Organization; 2001. Accessed April 13, 2023. https://www.who.int/classifications/icf/en/.

[40] Cieza A, Geyh S, Chatterji S, Kostanjsek N, Ustun B, Stucki G. ICF linking rules: an update based on lessons learned. J Rehabil Med. 2005;37:212–218. 10.1080/16501970510040263. 16024476

[41] Cieza A, Fayed N, Bickenbach J, Prodinger B. Refinements of the ICF linking rules to strengthen their potential for establishing comparability of health information. Disabil Rehabil. 2019;41:574–583. 10.3109/09638288.2016.1145258. 26984720

[42] Tuntland H, Aaslund MK, Langeland E, Espehaug B, Kjeken I. Psychometric properties of the Canadian Occupational Performance Measure in home-dwelling older adults. J Multidiscip Healthc. 2016;9:411–423. 10.2147/JMDH.S113727. 27621647 PMC5017582

[43] Horn KK, Jennings S, Richardson G, Vliet DV, Hefford C, Abbott JH. The patient-specific functional scale: psychometrics, clinimetrics, and application as a clinical outcome measure. J Orthop Sports Phys Ther. 2012;42:30–D17. 10.2519/jospt.2012.3727. 22031594

[44] Klokkerud M, Grotle M, Løchting I, Kjeken I, Hagen KB, Garratt AM. Psychometric properties of the Norwegian version of the patient generated index in patients with rheumatic diseases participating in rehabilitation or self-management programmes. Rheumatology (Oxford). 2013;52:924–932. 10.1093/rheumatology/kes401. 23335634

[45] Mokkink L, Terwee C, de Vet H. Key concepts in clinical epidemiology: responsiveness, the longitudinal aspect of validity. J Clin Epidemiol. 2021;140:159–162. 10.1016/j.jclinepi.2021.06.002. 34116141

[46] Rysstad T, Grotle M, Klokk LP, Tveter AT. Responsiveness and minimal important change of the QuickDASH and PSFS when used among patients with shoulder pain. BMC Musculoskelet Disord. 2020;21:328. 10.1186/s12891-020-03289-z. 32460743 PMC7254648

[47] Portney LG, Watkins MP. Foundations of Clinical Research: Applications to Practice. 3rd ed. Philadelphia: FA Davis Co; 2015.

[48] Koo TK, Li MY. A guideline of selecting and reporting intraclass correlation coefficients for reliability research. J Chiropr Med. 2016;15:155–163. 10.1016/j.jcm.2016.02.012. 27330520 PMC4913118

[49] Mokkink LB, Prinsen ACC, Patrick DL et al. COSMIN Methodology for Systematic Reviews of Patient-Reported Outcome Measures (PROMs). Amsterdam Public Health Research Institute. 2018.

[50] Giavarina D . Understanding Bland Altman analysis. Biochem Med (Zagreb). 2015;25:141–151. 10.11613/BM.2015.015. 26110027 PMC4470095

[51] Devji T, Carrasco-Labra A, Qasim A et al. Evaluating the credibility of anchor based estimates of minimal important differences for patient reported outcomes: instrument development and reliability study. BMJ. 2020;369:m1714. 32499297 10.1136/bmj.m1714 PMC7270853

[52] Terwee CB, Peipert JD, Chapman R et al. Minimal important change (MIC): a conceptual clarification and systematic review of MIC estimates of PROMIS measures. Qual Life Res. 2021;30:2729–2754. 10.1007/s11136-021-02925-y. 34247326 PMC8481206

[53] Turner BJ, Ownsworth TL, Turpin M, Fleming JM, Griffin J. Self-identified goals and the ability to set realistic goals following acquired brain injury: a classification framework. Aust Occup Ther J. 2008;55:96–107. 10.1111/j.1440-1630.2007.00660.x. 20887444

[54] Storløs B, Roaldsen KS, Soberg HL, Kleffelgaard I. Patient-specific functioning related to dizziness and balance problems after traumatic brain injury—a cross sectional study using an ICF perspective. Cog Med. 2021;8:1932247. 10.1080/2331205X.2021.1932247.

[55] Winters C, Kwakkel G, van Wegen EEH, Nijland RHM, Veerbeek JM, Meskers CGM. Moving stroke rehabilitation forward: the need to change research. NeuroRehabilitation. 2018;43:19–30. 10.3233/NRE-172393. 30056434

[56] Bernhardt J, Hayward KS, Kwakkel G et al. Agreed definitions and a shared vision for new standards in stroke recovery research: the stroke recovery and rehabilitation roundtable taskforce. Inter J Stroke. 2017;12:444–450. 10.1177/1747493017711816. 28697708

[57] Fitzpatrick R, Davey C, Buxton MJ, Jones DR. Evaluating patient-based outcome measures for use in clinical trials. Health Technol Assess. 1998;2:1–74. 10.3310/hta2140. 9812244

[58] Berdal G, Sand-Svartrud AL, Bo I et al. Aiming for a healthier life: a qualitative content analysis of rehabilitation goals in patients with rheumatic diseases. Disabil Rehabil. 2018;40:765–778. 10.1080/09638288.2016.1275043. 28084842

[59] Røe Y, Rysstad T, Tveter AT, Sandbakk TB, Jæger M, Grotle M. What are the most important problems in functioning among patients with shoulder pain? An analysis of the patient-specific functional scale. Phys Ther. 2021;101:pzab141. 10.1093/ptj/pzab141. PMC8485735 34089324

[60] Rutherford C, Costa D, Mercieca-Bebber R, Rice H, Gabb L, King M. Mode of administration does not cause bias in patient-reported outcome results: a meta-analysis. Qual Life Res. 2016;25:559–574. 10.1007/s11136-015-1110-8. 26334842

[61] Terluin B, Eekhout I, Terwee CB. The anchor-based minimal important change, based on receiver operating characteristic analysis or predictive modeling, may need to be adjusted for the proportion of improved patients. J Clin Epidemiol. 2017;83:90–100. 10.1016/j.jclinepi.2016.12.015. 28093262

[62] Brott T, Adams HP Jr, Olinger CP et al. Measurements of acute cerebral infarction: a clinical examination scale. Stroke. 1989;20:864–870. 10.1161/01.STR.20.7.864. 2749846

[63] Munthe-Kaas R, Aam S, Saltvedt I et al. Test accuracy of the Montreal Cognitive Assessment in screening for early poststroke neurocognitive disorder: the nor-COAST study. Stroke. 2021;52:317–320. 10.1161/STROKEAHA.120.031030. 33250039

[64] Rankin J . Cerebral vascular accidents in patients over the age of 60. III. Diagnosis and treatment. Scott Med J. 1957;2:254–268. 13432842

[65] Bobos P, MacDermid J, Nazari G, Furtado R, CATWAD. Psychometric properties of the global rating of change scales in patients with neck disorders: a systematic review with meta-analysis and meta-regression. BMJ Open. 2019;9:e033909. 10.1136/bmjopen-2019-033909. PMC6886942 31772112

[66] Chatman AB, Hyams SP, Neel JM et al. The patient-specific functional scale: measurement properties in patients with knee dysfunction. Phys Ther. 1997;77:820–829. 10.1093/ptj/77.8.820. 9256870

