# Supplementary material for: Measurement Properties of the Patient-Specific Functional Scale in Rehabilitation for Patients With Stroke: A Prospective Observational Study
Source: Phys Ther. 2023 Feb 13;103(5):pzad014. doi: 10.1093/ptj/pzad014 (PMC10158643; doi:10.1093/ptj/pzad014)
Supplement: Supplementary_Appendix_TSR_pzad014 [file supplementary_appendix_tsr_pzad014.pdf]

Appendix 1. Example of using the PSFS in collaborative development of rehabilitation goals

**1. Get to know the patients` preferences**<br>**Examples of questions asked:**<br>-Can you tell me about your life before you were diagnosed with a diagnosis of stroke?<br>-What did your everyday life look like?
**2. Develop long-term goals**<br>**Examples of questions asked:**<br>-What is important to you now in your situation with a diagnosis of stroke?<br>- What do you want to gain from rehabilitation?<br>**Examples of patients answers:**<br>-To be independent in daily routines.<br>-To be able to work again.
**3. Introduction of the PSFS**<br>-The health professionals informed the patients that we use the PSFS to measure the patients` opinion of their function and as a tool to develop collaborative rehabilitation goals.
**4. Develop short-term goals**<br>The health professionals guide the patients to transform the long-term goals into specific and shorter term goals, which could be an activity in the PSFS.<br>**Examples of questions asked:**<br>What activities are important to you but are challenging to perform due to your health condition?<br>What does it take to be able to perform this activity?

What will be the first thing you have to master to perform this activity?

Examples of patients answers:

Remember appointments, find my way at the mall, type text messages, complete online banking, have a conversation, walk upstairs, go to the store, go to the toilet alone, get dressed, clean the house, make dinner, go to the gym, join the grandchildren at a soccer game, knit.

Abbreviations: PSFS, Patient-Specific Functional Scale

Appendix 2. Examples of goals that were classified in the ICF components "Body Functions" and "Activities and participation"

Rehabilitation goals	Activities and participation	Body functions
To be able to write	d170 Writing	
To be able to walk	d450 Walking	
Communicate	d350 Conversation	
Cook	d630 Preparing meals	
Put on pants and socks without pain	d540 Dressing	b280 Pain
Remember messages and appointments		b144 Memory functions
Strengthen my hand to manage daily life	d230	b730 Muscle power functions
Walk to the mailbox and get the newspaper	d450 Walking / d210 Undertaking a single task	
Find words		b167 Mental function of language
Toileting on my own	d530 Toileting	
Train balance	d210 Undertaken a single task	b235 Vestibular functions
Use my left arm when I eat and drink	d445 Hand and arm use / d550 Eating	
See and manipulate small items such as screws and nails with my hands	d440 Fine hand use	b210 Seeing functions
Not to swallow wrong when I eat	d550 Eating	b5105 Swallowing
Play soccer with my grandchildren	d920 Recreation and Leisure	

Work 3 days in a week	d845 Acquiring, maintaining, and terminating a job	
